# Double Negative Is Positive: Anomalous Aortic Origin of the Left Coronary Artery Saved by a Bicuspid Aortic Valve

**DOI:** 10.7759/cureus.61136

**Published:** 2024-05-26

**Authors:** Anoohya Vangala, Sushmita Prabhu, Gaurav Mohan, Medha Ghose, Doantrang Du, Ajay Shah

**Affiliations:** 1 Internal Medicine, Monmouth Medical Center, Long Branch, USA; 2 Internal Medicine, Saint Vincent Hospital, Worcester, USA; 3 Cardiology, Monmouth Medical Center, Long Branch, USA

**Keywords:** sudden cardiac death, myocardial infarction, bicuspid aortic valve, aaoca, dyspnea

## Abstract

Atypical aortic origin of coronary artery (AAOCA) is a rare heart condition that has been identified in only a few autopsy cases and in some patients who have undergone angiographic evaluation. AAOCA is known to be closely linked with aortic valve malformations, with the most common type being the bicuspid aortic valve (BAV). A 77-year-old male with a medical history of hypertension and diabetes presented with dyspnea and orthopnea for three days. During the eventual cardiac catheterization, it was noted that the left coronary artery had an anomalous origin from the right side, coursing between the aorta and pulmonary artery. Contrast-enhanced computerized tomography (CECT) also showed a type 2 BAV and a left main coronary artery arising lower at the level of the pulmonary trunk. The dyspnea in this patient was attributed to diastolic dysfunction, and surgical correction of the coronaries was not done. The patient was managed on an outpatient basis for heart failure. In this case, the patient had an anomalous origin of the left coronary artery and type 2 BAV, which posed significant cardiovascular complications. It is unclear if the presence of the concomitant type 2 BAV led to the origin of the anomalous left coronary artery being at a lower level through its effect on the developmental mechanics. This lower origin may have resulted in lower compressive forces on the coronary artery as the inter-arterial pressures would be lower closer to the heart and farther from the lungs. Our case report aims to highlight this complex presentation where the BAV likely provides a benefit in AAOCA cases.

## Introduction

Atypical aortic origin of coronary artery (AAOCA) is a rare cardiac condition that has been identified in some autopsy cases (0.17%) and in some patients who have undergone angiographic evaluation (1.2%) [1]. Multiple variations of AAOCA have been documented over the years. The left main coronary artery can originate from the right sinus of the Valsalva or a separate ostium and can take different pathways, such as anterior to the pulmonary artery (anterior), posterior to the aorta (posterior), through the interventricular septum (intra-septal/intraconal), and between the aorta and pulmonary artery (inter-arterial) [2]. The inheritance of this congenital anomaly is yet to be elucidated. AAOCA is known to be closely linked with aortic valve malformations, with the most common type being the bicuspid aortic valve (BAV). Three types of congenital BAV have been identified in the literature, each with its own set of phenotypes. The three types are: (1) type I, which is a fused BAV with right-left cusp fusion, right-non cusp fusion, left-non cusp fusion, and an indeterminate phenotype; (2) type II, which is a 2-sinus BAV with latero-lateral and anteroposterior phenotypes; and (3) type III, which is a partial-fusion BAV type with a single phenotype characterized by a tiny raphe [3]. The pathogenesis of this association is largely attributed to genetic and environmental causes. Abnormal blood flow through the aortic valve during valvulogenesis may cause cusp separation failure [4]. BAV is a disease that affects the entire aortic root and can cause aortic root dilatation, aneurysms, and dissection. Coronary artery anomalies, along with BAV morphology, have been described as significant risk factors for coronary complications when undergoing aortic valve or root procedures [5]. However, the origin of the coronary arteries, followed by their coursing out of the heart, plays an important role in disease manifestation and subsequent management.

## Case presentation

**Figure 1:**
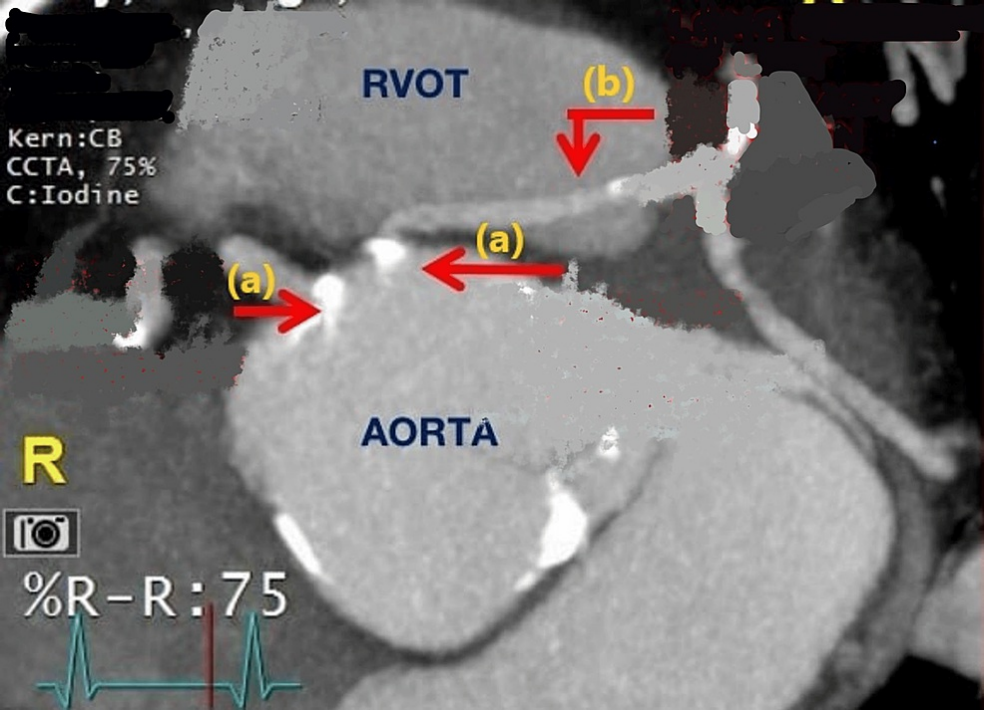
(a) Right and left coronary arteries arising from the anterior cusps and (b) left main coronary artery coursing between RVOT and the aortic root.

**Figure 2:**
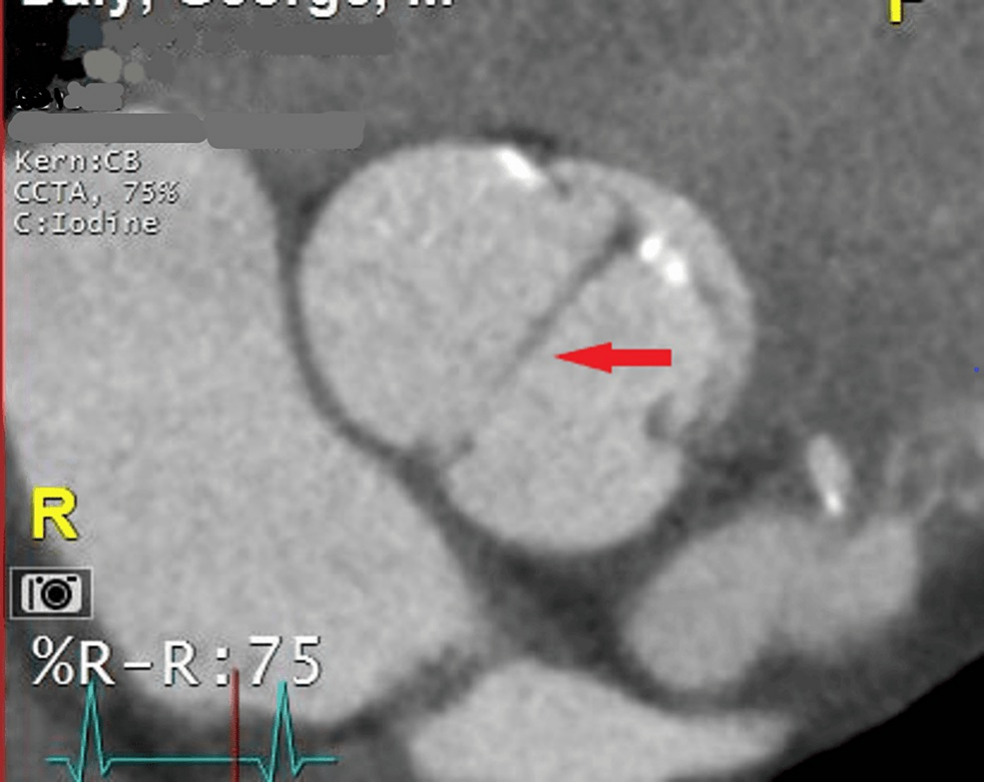
Red arrow showing an abnormal bicuspid aortic valve with a type 2 antral posterior commissure configuration.

A 77-year-old male with a medical history of hypertension and diabetes presented with dyspnea and orthopnea for three days. Vital signs were normal, and a physical exam revealed lower limb edema. Troponins and electrocardiography were normal. Echocardiography revealed an ejection fraction of 60-65%, grade 1 diastolic dysfunction, and moderate aortic stenosis. A contrast-enhanced computerized tomography (CECT) scan done to rule out pulmonary embolism revealed coronary artery calcification. During cardiac catheterization, it was noted that the left coronary artery had an anomalous origin from the right side, coursing between the aorta and pulmonary artery (Figure 1). CECT also showed a type 2 BAV and a left main coronary artery arising lower at the level of the pulmonary trunk (Figure 2). The dyspnea in this patient was attributed to diastolic dysfunction, and surgical correction of the coronaries was not done. The patient was managed as an outpatient for heart failure.

## Discussion

AAOCA is often discovered incidentally during a transthoracic echocardiogram or coronary angiographic study. However, it can have serious implications for myocardial circulation, which can lead to fatal complications such as myocardial infarction (MI) and sudden cardiac death (SCD) [6]. The cause of these complications is attributed to reduced coronary reserve caused by the oblique take-off from the aorta, ostial ridge, coronary vessel spasm, intussusception, noncompliant pericommissural area, and compression of the anomalous coronary artery intramurally and/or between the great vessels [7]. The best initial diagnostic modality, based on availability, cost-effectiveness, extent of invasion, and absence of radiation exposure, is a transthoracic echocardiogram with Doppler color flow mapping. Individuals without a history of ischemic chest pain or aborted SCD should undergo an exercise stress test combined with a nuclear perfusion scan to assess the ischemic burden. Moreover, computed tomography coronary angiograms (CTCA) and magnetic resonance coronary angiograms (MRCA) are more useful non-invasive techniques to identify coronary arteries and their anatomical course. A coronary angiogram can be performed in adults with risk factors for concomitant atherosclerotic coronary artery disease. Surgical intervention is typically necessary, including the unroofing of AAOCA if it is inter-arterial or intramural, pulmonary artery relocation, ostial translocation/reimplantation, osteoplasty, and bypass grafting [8].

In this case, the patient had an anomalous origin of the left coronary artery and type 2 BAV, which posed significant cardiovascular complications. It is unclear if the presence of the concomitant type 2 BAV led to the origin of the anomalous left coronary artery being at a lower level through its effect on the developmental mechanics. This lower origin may have resulted in lower compressive forces on the coronary artery as the inter-arterial pressures would be lower closer to the heart and farther from the lungs. This could decrease the likelihood of fatal complications at a young age and enable patients to live longer. 

The most recent expert consensus guidelines for AAOCA commissioned by the American Association of Thoracic Surgery (AATS) recommend that asymptomatic individuals with the left main coronary artery arising from the right sinus of Valsalva should be offered surgery (class 1, level of evidence B). However, there are many questions that remain unanswered in these guidelines, such as whether there is a lower or upper limit in terms of age for surgery and whether to perform corrective procedures if the anomaly is found incidentally at the time of surgery [9].

## Conclusions

Our case report aims to highlight this complex presentation where the BAV likely provides a benefit in AAOCA cases. Healthcare professionals should consider such exceptions when deciding on management strategies. Furthermore, more research is necessary to discern the possible mortality associations with BAV in concert with AAOCA.
